# Evaluation of Electronic Palliative Care Coordination Systems to support advance care planning for people living with life-threatening conditions (PREPARE): protocol for a multicentre observational study using routinely collected primary and secondary care data in England

**DOI:** 10.1136/bmjopen-2024-093175

**Published:** 2025-03-05

**Authors:** Marcel Alied, Sophie Law-Clucas, Matthew J Allsop, Christina Ramsenthaler, Peter May, Alastair Bearne, Margaret Powell, John Rosling, Rashmi Kumar, Lisa Scerri, Rebekah Williams, Katherine E Sleeman, Diane Laverty, Denzil James, Julia Verne, Kavitha Saravanakumar, Ceire E Costelloe, Joanne Droney, Jonathan Koffman

**Affiliations:** 1 Health Informatics, Division of Clinical Studies, The Institute of Cancer Research, London, UK; 2 Wolfson Palliative Care Research Centre, Hull York Medical School, University of Hull, Hull, UK; 3 Academic Unit of Palliative Care, Leeds Institute of Health Sciences, University of Leeds, Leeds, UK; 4 Cicely Saunders Institute of Palliative Care, Policy and Rehabilitation, King’s College London, London, UK; 5 Patient and Public Involvement Contributor, London, UK; 6 The Royal Marsden NHS Foundation Trust, London, UK; 7 Nurse Consultant, London, UK; 8 Office for Health Improvement and Disparities, London, Greater London, UK; 9 Director of Business Intelligence, NHS North West London, London, London, UK; 10 Imperial College London, London, UK

**Keywords:** PALLIATIVE CARE, Decision Making, Digital Technology, Electronic Health Records, EPIDEMIOLOGIC STUDIES, Health Services for the Aged

## Abstract

**Abstract:**

**Introduction:**

Electronic Palliative Care Coordination Systems (EPaCCS) are electronic registers that aim to facilitate documentation and sharing of up-to-date information about patients’ end-of-life preferences and plans for care among different health services. They aim to improve patients’ experiences and outcomes and mitigate costs linked to undesired aggressive care. However, evidence on the equitable delivery of EPaCCS and the extent to which advance care planning (ACP) enhances end-of-life care remains sparse. This study aims to explore the effect of EPaCCS on healthcare outcomes, service utilisation, and costs. It will also estimate the association between social determinants of health and the content and use of EPaCCS.

**Methods and analysis:**

The PREPARE project is a retrospective observational cohort study conducted in two phases. We will analyse routinely collected data from three EPaCCS registers from London, Bradford and Leeds. The first phase will use descriptive analysis to describe the completeness of EPaCCS, the content of EPaCCS, and socio-demographic and clinical characteristics of individuals with EPaCCS, and will model the relationship between social determinants of health and completion of ACP components and the creation of EPaCCS. The second phase will use a natural experiment to compare quality indicators (place of death and hospital use) between individuals with EPaCCS and those without. The control groups will be identified through the Leeds decedent dataset and through linking the London EPaCCS register to an electronic record used in North West London. Also, we will quantify healthcare costs and outcomes.

**Ethics and dissemination:**

Research approval has been secured from the Health Research Authority (ref 24/LO/0194), London - South East Research Ethics Committee (ref 24/LO/0194) and Confidentiality Advisory Group (ref 24/CAG/0046). Dissemination of findings will occur through peer-reviewed publications, knowledge exchange events and collaborative efforts with patient and public involvement partners.

**STRENGTHS AND LIMITATIONS OF THIS STUDY**

- Inclusion of all Electronic Palliative Care Coordination Systems (EPaCCS) records in London (2010–2022), Leeds (2015–2023) and Bradford (2015–2023) with an additional cohort of patients without EPaCCs for Leeds (2021–2023) and London makes this the largest collection of data sources in an EPaCCS UK study.
- Following the ‘nothing about us without us’ philosophy, there is strong patient and public involvement collaboration at every stage of the project.
- A natural experiment aims to provide credible causal estimates of EPaCCS effects on end-of-life outcomes, which will strengthen the evidence base.
- Recommendations to key stakeholders based on the findings will inform future EPaCCS use.
- Challenges comparing EPaCCS due to the interoperability of the data may limit the generalisability of the findings to other EPaCCs within the UK.

## Introduction

 Over 530 000 people die each year in the UK^1^ and this number is increasing.^2^ Many of these deaths are associated with people living with multiple morbidities and complex end-of-life care issues.^3^ The National Health Service (NHS) should provide high-quality, safe, person-centred end-of-life care consistent with their wishes.^4^ Most people wish to die at home.^5 6^ Additionally, hospital was the least preferred place of death for patients receiving palliative care in three countries.^6^ However, more than half of UK people currently die in hospital^2^ and many experience unplanned hospital admissions, including emergency department visits and unwanted and futile aggressive treatment.^27^,^9^ Failure to address these critical issues undermines the realisation of benchmarks that define a good death, as perceived by individuals, their families and healthcare providers alike.^10^,^12^

Advance care planning (ACP) is a voluntary process that supports adults in considering and sharing their values, goals and preferences regarding future care, including location of death so that if they lose mental capacity to make informed decisions for themselves, health professionals and their families can provide care consistent with their wishes.^13^ In the UK, ACP is endorsed in national policy.^14^ Despite scepticism of its value,^15^,^17^ an intrinsic logic of ACP underpins its use in practice and justifies continued research. Potential ACP benefits include providing important opportunities for discussion of diagnosis and prognosis so care and treatment are aligned with individuals’ preferences, improving symptom discussions and treatment adherence and reducing misunderstandings and conflict between medical staff and families.^18^ ACP may also lead to fewer interventions of limited or futile clinical value, earlier access to palliative care, reduced inappropriate emergency hospital admissions, fewer hospital deaths and increased rates of hospice admission or appropriate care at home.^19^,^21^ ACP is thought to help families prepare for the death of a loved one, resolve family conflict and help with bereavement.^22^ Although primarily concerned with improving the appropriateness and quality of care, ACP may contribute to controlling important health spending and making more appropriate and considered use of scarce resources in end-of-life.^18 23^

*The NHS Long Term Plan* advises building a ‘digital front door’ connecting health professionals to people^24^ to improve access, coordination, health outcomes and efficiency.^25^ In line with this, ACP records need to be accessible to healthcare professionals across different settings.

Electronic Palliative Care Coordination Systems (EPaCCS) have been specifically designed to facilitate seamless electronic information sharing and enable ACP and end-of-life care decision-making to increase the likelihood of delivering end-of-life care following patient wishes and priorities.^26^,^28^ EPaCCS records are intended for creation by trained healthcare professionals with input from patients and their carers to enable patients to discuss and make decisions about their preferences for end-of-life care such as preferred place of death, the ceiling of treatment and resuscitation status. Once stored, it is expected that information should be shared electronically with different professionals across different settings to inform decision-making, especially in times of crisis (emergency services (NHS 111 and 999), general practice, specialist palliative care services, hospices, etc).^26 27^ Nationally, 175 (83%) clinical commissioning groups (CCGs), now Integrated Care Boards (ICBs), have either implemented EPaCCS or started planning for their implementation.^29^ The Department of Health’s National Commitment for End-of-Life Care has recommended continued EPaCCS roll-out^30^ and they have been endorsed in the ‘Palliative and End-of-Life Care: Statutory Guidance for Integrated Care Boards (ICBs)’.^31^

While EPaCCS offer potential merits, no UK research has yet evidenced (1) to what extent EPaCCS support advance care planning, (2) to what extent EPaCCS have been offered equitably to all those who stand to benefit from them, (3) the effect of EPaCCS on patients’ place of death, and (4) if EPaCCS are associated with the use of health resources at the end of life where care costs are high.^32^

The PREPARE study therefore aims to (a) describe the characteristics of people who receive EPaCCS to support decision-making at the end of life and to estimate the association between social determinants of health and the content of ACP contained within EPaCCS and (b) explore EPaCCS effect on healthcare outcomes, use and costs. Based on our findings, we will synthesise recommendations on the use of EPaCCS to support end-of-life care for people living with life-limiting illnesses and their families.

The study objectives are the following:

1A. To describe and categorise the data fields and completeness of data contained within each EPaCCS register.

1B. To describe the creation and content of EPaCCS records and elements relating to end-of-life decision-making preferences and ACP (ceiling of treatment, preferred place of care, and death and resuscitation status) in different regions across England.
1C. To describe the socio-demographic and clinical characteristics of individuals who have created EPaCCS records in different regions across England.
1D. To estimate associations between social determinants of health (socioeconomic position, ethnicity, age and gender) and the completion of elements of ACP within EPaCCS.
1E. To estimate associations between social determinants of health (socioeconomic position, ethnicity, age and gender) and the creation of EPaCCS.
2A. To explore the effect of EPaCCS on place of death and hospital use in the last 90 days of life.
2B. To explore the effect of an EPaCCS record on healthcare costs in the last 90 days of life.

This HRA-approved protocol (V.1.1; January 2025) outlines in detail the motivation for this study, data sources, analysis plan, public and patient involvement, and ethical considerations.

### Methods and analysis

### Study design

Objectives 1A–1C will be addressed using descriptive statistics, objectives 1D and 1E using multivariable regression and objectives 2A and 2B using a natural experiment framework, which will allow us ultimately to make conclusions and recommendations about the continued use of EPaCCS.^33 34^ Descriptive statistics and multivariable regression are key tools in quantitative end-of-life research where there is a high reliance on routine data.^35^ However, these methods are not always reliable for generating credible causal estimates of treatment effects.^36^ Provided underlying assumptions are met,^37^ natural experiments can generate causal evidence from observational data, including routinely collected data. These have been little used in end-of-life care research to date,^38^ but may be particularly impactful in a field where randomised trials are infrequent and methodologically challenging,^39^ reliance on routine data is paramount^40^ and selection bias in routine data is a persistent concern.^41^

This evaluation of EPaCCS encompasses two distinct work packages that draw upon data from various routinely collected sources, and it is planned to be completed between 2024 and 2025. The first work package (WP1) will enable a comprehensive evaluation of the structure and completeness of three EPaCCS registers (London, Leeds and Bradford), the creation and content of EPaCCS records, exploring the social determinants of health of individuals with EPaCCS records and their association with the completion of elements of ACP within EPaCCS, and exploring the association of social determinants of health with EPaCCS creation. The second work package (WP2) will evaluate the benefits of care for individuals with EPaCCS records against those without. Additionally, it will investigate to what extent EPaCCS lead to better utilisation of scarce health resources. While the analysis of the two work packages will be conducted separately, understanding the content and structure of the datasets and the completeness of the data fields for the WP1 objectives will inform further analyses in WP2. Additionally, understanding which social determinants of health are associated with the creation of EPaCCS records will improve the analysis of WP2 by understanding how these variables should be treated in the statistical models. Together, the findings of WP1 and WP2 will provide evidence as to whether EPaCCS offer benefits to patients at the end of life, their families and the NHS. The study will derive high-quality evidence on factors influencing the uptake of EPaCCS, as well as the effect of EPaCCS on the costs and quality of end-of-life care.^42^

### Data sources

The study will use non-identifiable data from three EPaCCS across three distinct geographical locations in the UK, namely London, Leeds and Bradford, which combined have a population of 10.4 million people. These EPaCCS were introduced at different times within the same city and across different cities. This has led to natural variation in exposure, resulting in datasets that cover varying time periods: 2010–2022 in London, and 2015–2023 in Leeds and Bradford. Nonetheless, these EPaCCS are well established and contain a sufficient number of records, as illustrated in table 1, to enable meaningful individual site analyses and answer our research questions. Additional anonymised EPaCCS data from different regions will be considered if made available during the study. The sample size will be determined by the data available in the three datasets and will vary depending on the analysis.

**Table 1:** EPaCCS and data availability

EPaCCS location	EPaCCS specific data
London EPaCCS	Coordinate My Care (CMC) served as the commissioned EPaCCS in London from 2010 to 2022, during which time it was also the largest UK EPaCCS. Covering 32 CCGs serving a population of 8.9 million, this EPaCCS dataset reflects a diverse urban patient population in terms of diagnosis, ethnicity and socioeconomic background. CMC records comprise data from various healthcare settings, including primary and secondary care, hospices and nursing homes. The dataset encompasses demographics, diagnoses, care preferences, the ceiling of treatment, resuscitation status, living circumstances, prognosis, performance status, date and place of death. Additionally, data is available regarding how often the record was accessed by urgent and non-urgent healthcare workers. In later years, patients had the option of starting a ‘MyCMC’ plan, whereby they use a patient portal to input some data themselves. The anticipated numbers for inclusion in the dataset are 140 000 records, of which 100 000 are for deceased patients.
North West London dataset	CMC records will be linked with the ‘Whole Systems Integrated Care’ (WSIC) dataset. WSIC is an electronic record used by healthcare professionals in North West London, which has a population of 2.4 million, to document essential information about patient care in the region (eight health boroughs, previously defined as CCGs).^65^ The WSIC dataset will include all individuals who have died in North West London within the timeframe spanning from 2010 to 2023. This linkage will allow the creation of two cohorts for comparison: individuals with EPaCCS (CMC) and those without EPaCCS. All decedents with a CMC record within the WSIC catchment area will be identified for inclusion within the linked dataset, while decedents without such records will form the control group. The WSIC dataset includes coded primary, secondary, acute, mental health, community health and social care data. This dataset also includes detailed information on social determinants of health, including ethnicity, and mortality data, including place of death. Cost data is also available as patient-level costs referring to the indicative spend calculated separately for each patient for each healthcare sector. This will be used in a cost minimisation analysis of EPaCCS, and it includes primary care level, acute, community, mental health and social care costs. Data linkage will be carried out by North West London Integrated Care Board and supported by existing data sharing framework.^66^ Figure 1 illustrates the data flow and linkage process. The linked dataset will be de-identified prior to being made available to the research team for the analysis
Leeds EPaCCS	The Leeds EPaCCS dataset represents a combined dataset from community palliative care providers that includes all primary care practices and two city hospice sites (St Gemma’s Hospice and Wheatfields Hospice) and limited secondary care data (eg, unplanned hospital admissions in the last 90 days of life). This dataset encompasses decedent patient records spanning from 2015 to 2023. It includes linked healthcare records data including primary and secondary care data, such as hospital admissions and diagnosis of severe mental illness or learning disability. The EPaCCS in Leeds covers the entirety of the former Leeds CCG serving a population of around 870 000 people through 94 general practitioners (GP) practices. Since 2019, approximately 50% of all people who die in Leeds have an EPaCCS.^67^ Data for all deaths across Leeds from 2021 to 2023 are also available, including decedents without EPaCCS. This will enable comparative analysis between patients with or without EPaCCS records. The anticipated number of records for inclusion is 15 500 decedent records.
Bradford EPaCCS	The Bradford EPaCCS dataset is collected and shared among healthcare providers (including both NHS and voluntary sector providers) through electronic health records. This dataset includes decedent patient records spanning from 2015 to 2023. This EPaCCS is commissioned across the Bradford and Airedale districts, serving a combined population of 585 000 with approximately 4800 deaths per year. Approximately 48% of all deaths in this area had an EPaCCS record in 2018/19.^68^ The anticipated number of records for inclusion is 17 000 decedent records.

**Figure 1:**
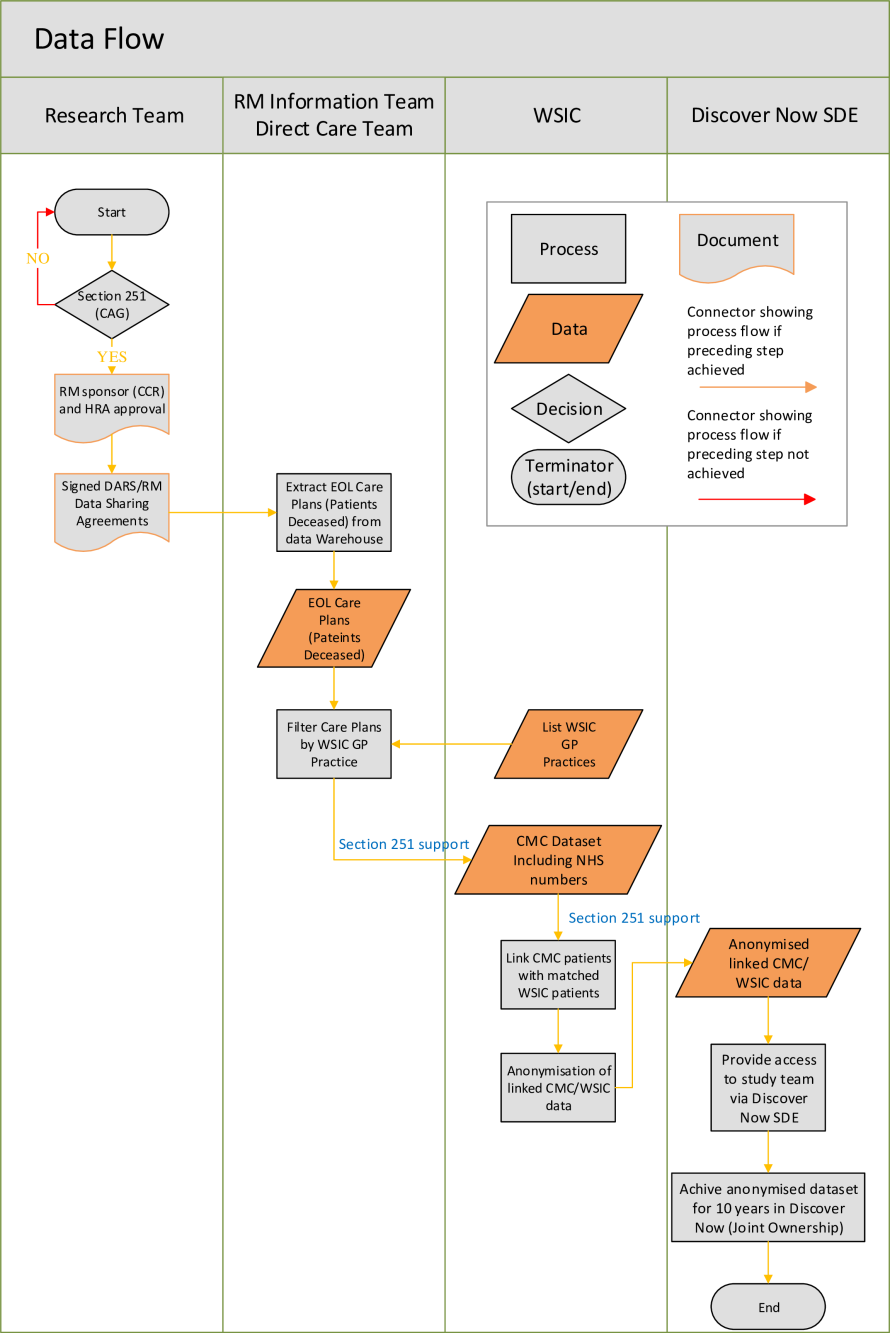
The data flow and the linkage of the Coordinate My Care dataset to the Whole System Integrated Care dataset. CAG, Confidentiality Advisory Group; CCR, Committee for Clinical Research; CMC, Coordinate My Care; DARS, Data Access Request Service; EOL, end-of-life; HRA, Health Research Authority; RM, Royal Marsden; SDE, secure data environment; WSIC, Whole Systems Integrated Care.

### Work package 1: exploration of EPaCCS records and association of social determinants of health in decision-making at the end of life (objectives 1A–1E)

In the first work package, a retrospective observational study, we hypothesise that this exploratory work package will help us understand the implementation of EPaCCS in terms of who creates EPaCCS and how EPaCCS are used, identify variation in their use and uptake, and clarify what information is included in these records.

The study will describe how EPaCCS are used, specifically who creates EPaCCS records and what information they encompass regarding individuals’ preferences for end-of-life care. The study will describe data fields across each of the three datasets and evaluate their completeness. Additionally, a descriptive analysis of the three EPaCCS datasets will be undertaken to describe the creation and content of the records across the three regions. These specific regions were chosen for being large metropolitan areas with varied levels of material deprivation and a high concentration of ethnic minority groups. This analysis will focus on the elements of ACP used to support decision-making at the end of life, which include documentation of the ceiling of treatment, resuscitation status, preferred place of care and preferred place of death. The study will also explore if changes are made to decision-making over time for each of these elements. As the three EPaCCS in the study contain data from different periods in time, we will take into account the different timescales during the analyses. The uptake of EPaCCS will be evaluated by describing the number of records created across different time periods and locations. The study will also investigate the setting of the initial record creation (hospital care, primary care, community care). Furthermore, the study will describe the clinical characteristics of patients such as their diagnosis, performance status, capacity for decision-making, living arrangements and expected prognosis. The study will also describe their socio-demographic characteristics with a particular emphasis on social determinants of health such as age, gender, socioeconomic position and ethnicity. The socioeconomic position will be defined using the Index of Multiple Deprivation (IMD), a standard scoring system based on a range of economic, social and housing data, creating a single deprivation score for each small area of the country. Using methodology previously employed in studies by the research team,^43 44^ an IMD score will be created for each patient based on their postcode which will reflect the deprivation data for the Lower-layer Super Output Area (LSOA) within which the postcode falls. Moreover, the study will investigate across sites the association between social determinants of health and the completion of each of the four previously mentioned ACP elements. Table 2 shows the mapping of the study objectives to data sources and eligibility criteria. Exclusion criteria for all data sources are participants who have chosen National Data Opt-Out and those who have withdrawn consent.

**Table 2:** Mapping study objectives to data sources and eligibility to be included in analyses

Objectives		Data sources and eligibility
Work package 1		
1A	To describe and categorise the data fields and completeness of data contained within each EPaCCS register.	Anonymised EPaCCS data from London (CMC, n=140 000), Leeds (n=15 500) and Bradford (n=17 000)
1B	To describe the creation and content of EPaCCS records and elements relating to end-of-life decision-making preferences and ACP (ceiling of treatment, preferred place of care, and death and resuscitation status) in different regions across England.	
1C	To describe the socio-demographic and clinical characteristics of individuals who have created EPaCCS records in different regions across England.	
1D	To estimate associations between social determinants of health (socioeconomic position, ethnicity, age, and gender) and the completion of elements of ACP within EPaCCS.	
1E	To estimate associations between social determinants of health (socioeconomic position, ethnicity, age, and gender) and the creation of EPaCCS.	WSIC-CMC dataset of North West London deceased patients with/without EPaCCSLeeds decedent dataset 2021–2023 of deceased patients with/without EPaCCS
Work package 2		
2A	To explore the effect of EPaCCS on place of death and hospital use in the last 90 days of life.	WSIC-CMC dataset of North West London deceased patients with/without EPaCCSLeeds decedent dataset 2021–2023 of deceased patients with/without EPaCCS
2B	To explore the effect of an EPaCCS record on healthcare costs in the last 90 days of life.	WSIC-CMC dataset of North West London deceased patients with/without EPaCCS

### Analysis

The data from each of the three distinct EPaCCS will be analysed separately. Individual patient-level EPaCCS data will be accessed and cleaned for each of the three systems. The study will categorise the data into social determinants of health (age, gender, ethnicity, social deprivation), clinical characteristics and elements relating to ACP. The social determinants of health have been identified from existing literature for being associated with poor health experiences and outcomes. The study will assess the completeness of variables in the data, examining patterns of missing data at the individual level and identifying any outlier records. Based on the characteristics of missing data, the study will adopt an appropriate approach to treating missing data for statistical analysis. Several methods will be considered that are appropriate for missing at random and not missing at random data. Though, it is assumed that the missing mechanism will be missing not at random. However, no robust methods of imputing missingness in the independent variables with a missing not at random mechanism have been identified. If imputation was carried out, a sensitivity analysis will be conducted to compare parameter estimates with/without imputation. Missing values for key outcomes will be imputed using gender, age, comorbidity, cause of death, IMD and/or study area.^45^ For each ACP element, documented decisions will be categorised to create an outcome (binary or ordinal) variable for inclusion in logistic regression modelling of the relationship between each of these outcomes, as distinct analyses, and social determinants of health, adjusting for major confounding variables and consideration of random effects/fixed effects (eg, comorbidity). Confounders will be selected using expert knowledge, existing literature, and the completeness and availability of the data. Using the London and Leeds datasets, a dichotomised binary outcome of whether or not a patient has an EPaCCS record will be created for logistic regression, modelling the association between the creation of EPaCCS records and social determinants of health. The study will identify both similarities and differences between the creation, use and content of EPaCCS records in the three EPaCCS cohorts. Mindful of the reported challenges associated with the interoperability of EPaCCS data,^46^ the comparison of datasets will be cautiously approached. We will examine the impact of the significant increase in EPaCCS records in London,^47^ and potentially the two other cities, following the outbreak of COVID-19 in March 2020 in terms of the documentation and completeness of ACP elements within the EPaCCS records. We will carry out a secondary analysis (sensitivity analysis) excluding individuals whose records were created during the COVID-19 pandemic after examining the data and the trend in the creation of EPaCCS records.

### Work package 1 outputs

WP1 will provide evidence of the variation in the uptake of EPaCCS across the three regions in the UK and will identify factors associated with unequal EPaCCS access. The study will describe similarities and differences in the content and creation of EPaCCS which will inform the development of standardised EPaCCS in the future. We will work with key stakeholders (EPaCCS providers, ICBs and health professionals) and in close collaboration with local populations to ensure those who promote end-of-life care decision-making facilitated by EPaCCS are more accessible and acceptable to individuals across the social strata. This will also include bespoke training for health professionals in culturally competent and literate care to enable end-of-life care discussions and decision-making.

### Work package 2: evaluation of the effect of EPaCCS on place of death and secondary healthcare use at end of life and cost minimisation analysis of EPaCCS (objectives 2A and 2B)

The second work package will adopt a retrospective cohort design under a natural experiment framework to evaluate the effects of EPaCCS on quality indicators of end-of-life care. The quality indicators used in this work package include the place of death (primary outcome) and time spent in hospital in the last 90 days of life (secondary outcome).^48^ The hypothesis for work package 2 is that people with EPaCCS records are less likely to die in hospital and more likely to spend less time in hospital in the last 90 days of life compared with people without EPaCCS records. An EPaCCS record may address one or both sides of the cost-effectiveness ratio; reducing futile or unwanted treatments will save hospital resources while improving goal-concordant care realises more utility. However, there is also the potential for increased costs, notably through substitution effects, for example, if people die in a hospice instead of in a hospital. We therefore hypothesise that EPaCCS improve the cost-effectiveness of care near the end of life.

Using the CMC-WSIC linked dataset and the Leeds dataset, the study will use a natural experiment approach^33^ and establish cohorts of patients who have died having EPaCCS (case) or not having an EPaCCS (control) in each dataset separately. Place of death will be coded as a binary variable for individual-level analysis, indicating whether death occurred in a hospital or elsewhere. For ecological analysis, rates of hospital deaths per year will be calculated for each one of the eight health boroughs (previously called CCGs) in North West London. Time spent in hospital will be derived as a continuous variable measuring the number of days between admission and discharge within the last 90 days of life, or between admission and death for patients who died in hospitals. Rates of time spent in hospital within the last 90 days of life per year will also be calculated. The cost analysis will use the patient-level cost data available in WSIC. Formal costs will be estimated by combining utilisation frequencies in the data with unit costs for different services.^49^ Informal care hours will be estimated from the literature on end-of-life care populations and associated costs using the substitution method (primary analysis) and opportunity cost method (sensitivity analysis). Intervention costs will be estimated using NHS data.^50^ We will model costs after diagnostic testing of different modelling approaches in the context of distributional characteristics.^51^

### Creating comparative cohorts

This study will take an inclusive approach to defining the cohorts for inclusion in the individual-level analysis, identifying deceased patients who had mention of one of the four main disease-related causes of death in their clinical records within the last year of life (cancer, dementia, heart or lung disease). Based on previous analyses^52^ and publicly available national data,^53^ we hypothesise that 22–30% of the EPaCCS cohort will die in the hospital compared with 45–50% in the non-EPaCCS group. The inverse probability of treatment weighting will be used to account for the confounding resulting from the non-random allocation of EPaCCS to the groups and balance characteristics between cohorts.^54^ First, a multivariable logistic regression will be used to determine the propensity score weights. Factors included in the model to estimate propensity scores will include the confounders selected to be included in the main outcome analysis (such as age, sex, ethnicity, deprivation and primary diagnosis). These confounders will be cautiously selected based on thorough discussions with our team, which includes experts in palliative care, input from the patient and public involvement group with lived experience in end-of-life care, and a comprehensive literature search. Then, for each patient inpatient encounter, a weight defined as the inverse of the probability of the treatment they had received will be calculated. The balance of patients’ characteristics will be assessed visually and by examining the standardised mean differences.^55^

### Ecological analysis

Treating the regions covered within the North West London ICBs as a random effect, mixed effects regression will be used to examine the effect of the rate of patients who died having EPaCCS on the annual rates of hospital deaths and time spent in hospital within the last 90 days of life, calculated, respectively, as the number of deaths with EPaCCS over total deaths, the number of in-hospital deaths over total deaths, and the aggregate time spent in hospital within the last 90 days of life over total deaths.^56^ Other approaches will be considered upon examining the data and the annual number of patients who died having EPaCCS.^33^

### Individual patient-level analyses

Mixed effects logistic regression will be used to assess the effect of EPaCCS on the primary and secondary outcome measures adjusting for covariates associated with these outcomes such as the availability of carers, social determinants of health (age, gender, ethnicity, social deprivation), marital status, living circumstances, place of care and comorbidity. To account for the time-varying nature of implementation, the season/financial quarter will also be included in the model. Marginal ORs for each of the outcomes based on weighted logistic regression will be reported.

### Missing data considerations

Individual-level missing data of the primary outcome variable (place of death) will be excluded from the multivariable analysis. However, in handling missing data related to covariates, those with incomplete data will be identified for a thorough characterisation of the missingness. Depending on the type of data and the level and mechanism of missingness, individual patients without matching covariate data may be excluded from the multivariable modelling. In doing so, we would also be mindful that by excluding such patients, modelling could be subject to biases, for example, due to under-reporting. Hence, we may consider imputing missing data, with sensitivity analysis to determine the effectiveness of imputation on the model outputs.

### Sensitivity analysis

We will consider a number of approaches in terms of sensitivity analyses, which will be informed by the availability and completeness of the data.^57^ Sensitivity analyses will be conducted on the cohort identification strategy and our choice of covariates used for estimating the propensity scores.^58^ We will also consider several approaches and sensitivity analysis techniques to deal with unobserved confounding, the implementation of new policies and the effect of the COVID-19 pandemic on both the intervention and the outcomes of interest.^33 59^ In terms of the COVID-19 pandemic, we will build on our previous evaluation,^47^ identify the records created during the pandemic and, if appropriate, perform a sensitivity analysis by potentially excluding these records to assess the impact of this period on our findings.

### Outputs from work package 2

The second work package will provide novel insight into the effect and value of EPaCCS and ACP on end-of-life care quality outcome measures. We will derive high-quality evidence on how the intervention affects costs in a context where a trial is unfeasible. Secondary analysis will provide insights into how end-of-life care interventions may address or exacerbate inequities in the context of systematic gaps.

### Reporting guidelines

The data analysis in this study will be guided by the Reporting of Studies Conducted using Observational Routinely-Collected Health Data extension to the Strengthening the Reporting of Observational Studies in Epidemiology guidelines.^60^ A full statistical analysis plan will be developed.

### Patient and public involvement

The study places a significant emphasis on patient and public involvement (PPI) to ensure its relevance for individuals with life-limiting conditions and their families. Key contributors include patients, caregivers and those with prior PPI roles, who have actively participated in various stages of previous research projects. Their involvement has extended from project conception to data analysis and dissemination. PPI coapplicants will improve this study by promoting accountability, appropriateness and advocacy, and alerting networks to findings. They played a role in presenting the study to a patient and carer research forum, receiving endorsement for its significance. Their ongoing participation expands from contributions to study development to interpretation and dissemination of findings. Tailored training and support will be provided for our PPI colleagues to ensure their meaningful contributions to the interpretation of the findings and the formulation of clinical and research-based recommendations.

## Ethics and dissemination

### Ethical considerations

The PREPARE study adheres to the principles of Good Clinical Practice, Data Protection Regulations, the Data Protection Act 2018 and other regulatory requirements in handling confidential patient information. The Leeds and Bradford datasets are de-identified patient-level data from GP practices with data sharing agreements in place for the use of this information in research to improve overall patient care. Explicit consent for Leeds and Bradford is not obtained, as there is an existing data agreement with all GP practices in those regions to use patient data to improve overall care, which covers the data used in this study. The use of both datasets is exempt from the Research Ethics Committee (REC) review, as these use only anonymised patient data routinely collected in the course of normal care.^61^ However, the use of identifiable patient information without consent in the linkage of CMC and WSIC data requires approval from both the REC and the Confidentiality Advisory Group (CAG). Research approval has been secured from the Health Research Authority (ref 24/LO/0194), London - South East REC (ref 24/LO/0194) and CAG (ref 24/CAG/0046). Patients actively consented to the use of their anonymised information for research at the time of creating a CMC record. Since the individual patients are deceased, explicit consent for linking the CMC and WSIC data is not possible. Nonetheless, given the cost of delivering EPaCCS and the need for evidence to support their roll-out, the public interest in the outcome of this research justifies the controlled use of individuals’ confidential information without gaining their consent. The flow of EPaCCS data to North West London ICB for data linkage will be carried out by the Royal Marsden Information Team, a trusted third party. Stringent measures, including secure file transfer protocols, Data Protection Impact Assessment and encryption are in place to protect the confidentiality of data. Subsequently, the linked datasets will be de-identified, ensuring the separation of Personal Identifying Information from analysis variables. Data will not be made available to the researchers until all necessary approvals are in place. The data will only be accessible by MA, SL-C, MJA, CR, PM, LS, CEC, JD and JK through Trusted Research Environments (TRE). TREs are secure research platforms that are designed to store and protect sensitive data, guaranteeing data privacy.^62^ Anonymised data for Leeds and Bradford will be stored and analysed in LASER (the Leeds Analytic Secure Environment for Research^63^), for London CMC in the Royal Marsden BRIDgE TRE (Biomedical Research Informatics Digital Environment^62^) and for the linked CMC-WSIC data in WSIC TRE (Whole Systems Integrated Care Trusted Research Environment aka Discover Now SDE (Secure Data Environment)^64^).

We carried out an Equality Impact Assessment with our PPI colleagues, where we acknowledged the importance of ensuring adequate diversity within our research team (including PPI team) and having adequate diversity of interests within the team. Based on the results, we will work with targeted community connectors to shape our patient-facing outputs and disseminate the findings.

The study has been peer-reviewed as part of the sponsorship approval process by the Royal Marsden and Institute of Cancer Research Committee for Clinical Research, which ensures the validity of our research. The study also benefited from expert input from senior researchers. Amendments to the original protocol will be subject to the sponsor’s approval and determination of whether amendments are substantial or not. Substantial amendments that require approval will only be implemented after approval. Any deviation from the protocol will be documented and reported.

### Dissemination plan

Our dissemination strategy aims to reach diverse audiences, engaging patients, caregivers, healthcare professionals, policymakers and researchers to influence clinical care and health policy nationally and internationally. Through collaboration with our PPI representatives, lay summaries of the research findings will be generated to engage a broad audience. We are also mindful of sharing the findings with lay, non-professional audiences and among ethnically diverse communities where data were collected. The findings will be shared electronically and through presentations to patient and caregiver groups. We will use social media platforms and actively participate in annual research open days and public engagement events to present aspects of the study. We anticipate the study findings will be of considerable interest to policymakers, particularly since EPaCCS are a proposed model to improve integration and personalised care, a feature central to Integrated Care Systems. Policymakers will receive findings through evidence summaries, policy briefings and participation in relevant conferences. Health professionals will be engaged through virtual workshops, offering insights into study findings and sharing ‘study finding briefs’ to stimulate discussions. Academic contributions will be made through publications in reputable journals and presentations at conferences. The ‘EPaCCS Research Network (ERN)’ will also be well placed to explore collaboration through follow-on funding to address gaps in evidence and to develop interventions for optimising engagement with EPaCCS identified during this current study. At the end of this study, we will make the anonymised CMC and linked anonymised CMC-WSIC data used in this study available through the Health Data Research UK (HDR-UK) Innovation Gateway to enable the preservation, sharing and reuse of data in other studies.

## Data Availability

Data are available upon reasonable request. Data may be obtained from a third party and are not publicly available.
