# Supplementary material for: Evaluation of Electronic Palliative Care Coordination Systems to support advance care planning for people living with life-threatening conditions (PREPARE): protocol for a multicentre observational study using routinely collected primary and secondary care data in England
Source: BMJ Open. 2025 Mar 5;15(3):e093175. doi: 10.1136/bmjopen-2024-093175 (PMC11883539; doi:10.1136/bmjopen-2024-093175)
Supplement: Supplementary file 1 [file bmjopen-2024-093175.reviewer_comments.pdf]

# PEER REVIEW HISTORY

BMJ Open publishes all reviews undertaken for accepted manuscripts. Reviewers are asked to complete a checklist review form and are provided with free text boxes to elaborate on their assessment. These free text comments are reproduced below.

## ARTICLE DETAILS

### Title (Provisional)

Evaluation of Electronic Palliative Care Coordination Systems to support advance care planning for people living with life-threatening conditions (PREPARE): protocol for a multicentre observational study using routinely collected primary and secondary care data in England

### Authors

Alied, Marcel; Law-Clucas, Sophie; Allsop, Matthew; Ramsenthaler , Christina; May, Peter; Bearne, Alastair; Powell, Margaret; Rosling, John; Kumar, Rashmi; Scerri, Lisa; Williams, Rebekah; Sleeman, Katherine E; Laverty, Diane; James, Denzil; Verne, Julia; Saravanakumar, Kavitha; Costelloe, Ceire E; Droney, Joanne; Koffman, Jonathan

---

## VERSION 1 - REVIEW

---

| | |
|---|---|
| **Reviewer** | **1** |
| **Name** | **Metallinou, Dimitra** |
| **Affiliation** | **University of West Attica, Midwifery** |
| **Date** | **18-Nov-2024** |
| **COI** | **None** |

---

Thank you for the opportunity to review this interesting and insightful study protocol. I would like to offer some suggestions and comments that I hope will help to improve the clarity, structure, and overall quality of the manuscript/protocol. I have attached a file containing detailed comments for your review.

| **Journal** | BMJ Open |
|---|---|
| **Manuscript ID** | bmjopen-2024-093175 |
| **Manuscript Title** | Evaluation of Electronic Palliative Care Coordination Systems to support advance care planning for people living with life-threatening conditions (PREPARE): protocol for an observational study using routinely collected data |

| |
|---|
| Thank you for the opportunity to review this interesting and insightful study protocol. I would like to offer some suggestions and comments that I hope will help to improve the clarity, structure, and overall quality of the manuscript/protocol. |
| **Summary and Scope**<br>This study protocol aims to evaluate the impact of Electronic Palliative Care Coordination Systems (EPaCCS) on advance care planning (ACP) for individuals with life-threatening conditions. The research design is a retrospective observational cohort study using data from three regions in the UK: London, Leeds, and Bradford. The study focuses on assessing the completeness, usage and effectiveness of EPaCCS records in supporting end-of-life care, analyzing outcomes like place of death, healthcare utilization and cost implications, and examining social determinants of health associated with ACP uptake and outcomes. |
| **Administrative Information**<br>1. *Protocol Version:* The study protocol does not include a version identifier or date. Including a protocol version (e.g., version 1.0, date, details: initial version etc.) is important for documenting the development of the protocol and ensuring transparency. This information is critical for tracking any future modifications and aligning with SPIRIT guidelines. Please add a protocol version and relevant details at the beginning of the document.<br><br>2. *Roles and Responsibilities:* While the protocol includes information about the approval process and amendment procedures, it lacks clarity regarding the funders' roles in specific study activities. Please clearly state whether the funders have any influence on the study design, data collection, management, analysis etc. Including a statement clarifying the extent of funders' involvement - or lack thereof - would enhance transparency, align the protocol with best practices, and ensure there is no perceived conflict of interest. |
| **Introduction**<br>It would be valuable to clearly state the primary and secondary aims and hypotheses of the study. |
| **Methods**<br>1. *Study design (Line 166):* First, please clearly specify the study design and provide detailed information on the setting and locations. The manuscript describes a "mixed-methods retrospective approach", incorporating elements of both observational and cohort study designs. However, it does not clearly distinguish how these approaches are applied across the two work packages. To improve clarity, please explicitly state that Work Package 1 (WP1) is a retrospective observational study focusing on data completeness, content, and associations with social determinants of health. In contrast, Work Package 2 (WP2) adopts a retrospective cohort design under a natural experiment framework to evaluate causal relationships. This distinction will enhance the clarity of the methodological |

approach and ensure consistency with the study's stated objectives. Additionally, the manuscript refers to a natural experiment framework but provides limited detail on the natural variation that determines exposure (e.g., the availability or implementation of EPaCCS).

2. *Participants*: While the protocol does not explicitly classify "eligibility criteria," authors define the population and data included in the analyses. The authors could strengthen the protocol by labeling these aspects as "eligibility criteria" and systematically outlining inclusion and exclusion factors in a dedicated section. Please also consider adding a subsection titled "Participants", where you can detail the eligibility criteria, the sampling method employed, and the sample size. These would enhance clarity and rigor to the protocol.

3. *Analysis:* Ensure that all information related to statistical analysis is relocated to the appropriate Statistical Methods subsection for better organization**.** To further strengthen the robustness of the analysis, it is recommended that authors include advanced methods designed to address confounding and bias, particularly in the context of natural experiments.

**Strengths**

1. *Significant Scope and Relevance:*
   - The study addresses an important and underexplored area of healthcare, focusing on the integration of digital technologies in palliative care.
   - The research has substantial potential to inform policy and clinical practice, particularly concerning equity, healthcare resource optimization, and alignment with patient preferences.

2. *Robust Dataset:*
   - The inclusion of three geographically and socioeconomically diverse regions, with over 120,000 anticipated records across various healthcare settings, ensures the study is well-powered to generate meaningful insights.
   - Linking datasets (e.g., Coordinate My Care and Whole Systems Integrated Care) to compare outcomes between EPaCCS and non-EPaCCS users adds significant depth to the analysis.

3. *Methodological Approach:*
   - The two-phase structure, combining descriptive statistics, multivariable regression and natural experiments, demonstrates methodological rigor and is well-suited for addressing both descriptive and causal research questions.

4. *Patient and Public Involvement:*

- The study includes strong patient and public involvement, aligning with the ethos of collaborative healthcare research and ensuring the relevance of findings for patients and families, credibility and impact of research.

5. *Ethical Considerations:*
   - The authors demonstrate an exemplary approach to ethical governance, with clear processes for data anonymization, secure handling, and appropriate approvals from regulatory bodies.

6. *Practical Implications:*
   - The findings have the potential to inform policy and practice, particularly regarding equity in access to EPaCCS, end-of-life decision-making, and healthcare cost-effectiveness.

**Limitations and Areas for Improvement**

Despite its strengths, the manuscript has some areas that require additional clarification and refinement. These include:

*1. Data Interoperability Challenges:*

- The variability in the completeness and interoperability of datasets across regions poses a risk to the comparability of results. The manuscript acknowledges this but does not adequately specify how these challenges will be mitigated during the analysis.

*2. Regional Variability:*

- The differences in healthcare delivery and ACP practices across London, Leeds, and Bradford could significantly influence findings. The protocol should outline how these regional differences will be statistically accounted for to ensure valid comparisons.

*3. Potential Selection Bias:*

- The observational nature of the study introduces inherent risks of selection bias. Patients with EPaCCS records may systematically differ from those without in ways that influence outcomes (e.g., severity of illness, access to healthcare). While inverse probability weighting is mentioned, further details on confounder selection and balance assessment are necessary.

*4. Clarification of Sensitivity Analysis:*

- The manuscript briefly mentions sensitivity analyses, especially regarding the COVID-19 pandemic's impact on EPaCCS documentation. More specific details on planned sensitivity analyses could clarify how the study intends to handle confounding factors.

*5. Cost Analysis Details:*

- While the study promises a cost minimization analysis, details on how costs will be attributed to specific healthcare services and modeled (e.g., inclusion of indirect costs, use of unit costs) are limited.

**Suggestions for Improvement**

1. *Enhance Description of Data Quality Management:* The protocol could benefit from a more detailed discussion on handling missing or incomplete data. The manuscript should specify the imputation methods, the thresholds for acceptable levels of missingness, and how sensitivity analyses will address potential biases introduced by missing data.

2. *Addressing Confounding:* The protocol should provide greater detail on how confounding variables will be selected and modeled. Including a directed acyclic graph (DAG) would enhance transparency in identifying causal relationships and addressing biases.

3. *Expound on Social Determinants Analysis:* The planned analysis of social determinants of health in relation to ACP and EPaCCS usage is a valuable component of the study. Expanding on the methodology for this analysis, including specific variables and adjustment methods, would improve transparency.

4. *Address Regional Differences:* A stronger discussion of regional differences in healthcare infrastructure, population demographics, and end-of-life care preferences would enhance the study's generalizability and relevance.

5. *Sensitivity Analyses:* The mention of sensitivity analyses is brief. The manuscript should elaborate on how these analyses will evaluate the robustness of findings, particularly in response to assumptions about confounders, selection bias, and the COVID-19 pandemic's impact on EPaCCS adoption.

6. *Ethics and dissemination:* While public access to datasets is reported, it is contingent on compliance with ethical and governance frameworks, ensuring secure and responsible use of sensitive health data. This aligns with best practices for handling healthcare data; however, greater clarity is needed regarding who will have the right to access the data or under what conditions access can be requested.

| **Scientific Impact**<br>The study has the potential to make a substantial contribution to the fields of palliative care, health informatics, and health policy. Its findings could provide actionable recommendations for optimizing EPaCCS implementation and highlight areas where equity in end-of-life care requires improvement. The focus on social determinants of health and the use of advanced statistical methods to infer causality further enhance its potential impact. |
| --- |
| **Conclusion**<br>This is a well-designed study protocol that addresses critical gaps in our understanding of the role of digital systems in end-of-life care planning. By implementing the recommendations above, the authors can further strengthen the rigor and clarity of their approach. The study is likely to yield valuable insights into improving end-of-life care, aligning healthcare delivery with patient preferences, and addressing inequities in access to palliative care resources. |

---

| | |
| --- | --- |
| **Reviewer** | **2** |
| **Name** | **Gonçalves, Florbela** |
| **Affiliation** | **University of Porto** |
| **Date** | **25-Nov-2024** |
| **COI** | **None** |

---

Thank you very much to the editors and authors for the opportunity to review this magnificent article.

It is very well written and innovative. I just have one question: how is data privacy guaranteed?

Good luck!

---

## VERSION 1 - AUTHOR RESPONSE

1. Dr. Dimitra Metallinou's comments:

| Comments | Modifications |
| --- | --- |
| Thank you for the opportunity to review this interesting and insightful study protocol. I would like to offer some suggestions and comments that I hope will help to improve the clarity, structure, and overall quality of the manuscript/protocol. I have attached a file containing detailed comments for your review. | We are deeply grateful for Dr. Metallinou's insightful comments and detailed review. Their feedback has been invaluable in improving the quality of the manuscript. We have addressed their comments in detail below. |

| | |
|---|---|
| Protocol Version: The study protocol does not include a version identifier or date.<br>Including a protocol version (e.g., version 1.0, date, details: initial version etc.) is important for documenting the development of the protocol and ensuring transparency. This information is critical for tracking any future modifications and aligning with SPIRIT guidelines. Please add a protocol version and relevant details at the beginning of the document. | We have included protocol version and date in the main text.<br><br>(P7, L168)<br>This HRA-approved protocol (version 1.1; January 2025) outlines in detail the motivation for this study, data sources, analysis plan, public and patient involvement, and ethical considerations. |
| 2. Roles and Responsibilities: While the protocol includes information about the approval process and amendment procedures, it lacks clarity regarding the funders' roles in specific study activities. Please clearly state whether the funders have any influence on the study design, data collection, management, analysis etc. Including a statement clarifying the extent of funders' involvement - or lack thereof - would enhance transparency, align the protocol with best practices, and ensure there is no perceived conflict of interest. | We have included a statement about funders involvement in the study.<br><br>(P21, L497)<br><br>The funders do not have a role in the study design, data collection, management or analysis. |
| **Introduction**<br>It would be valuable to clearly state the primary and secondary aims and hypotheses of the study. | As there are two work packages there are two primary aims, instead of a primary and a secondary aim. The text has been adjusted to say this, and the hypotheses for the two work packages have been included in their respective sections.<br><br>(P11, L220)<br><br>In the first work package, a retrospective observational study, we hypothesise that this exploratory research will help us understand the implementation of EPaCCS in terms of who creates EPaCCS, how EPaCCS are used, identify variation in their use and uptake and clarify what information is included in these records.<br><br>(P15, L306)<br><br>The hypothesis for work package 2 is that people with EPaCCS records are less likely to die in hospital and more likely to spend less time in hospital in the last 90 days of life compared to people without EPaCCS records. An EPaCCS record may address one or both |

| | |
|---|---|
| | sides of the cost-effectiveness ratio; reducing futile or unwanted treatments will save hospital resources while improving goal-concordant care realises more utility. However, there is also the potential for increased costs, notably through substitution effects, e.g. if people die in a hospice instead of in a hospital. We therefore hypothesise EPaCCS improve the cost-effectiveness of care near the end of life. |
| **Methods**<br>1. Study design (Line 166): First, please clearly specify the study design and provide detailed information on the setting and locations. The manuscript describes a “mixed-methods retrospective approach”, incorporating elements of both observational and cohort study designs. However, it does not clearly distinguish how these approaches are applied across the two work packages. To improve clarity, please explicitly state that Work Package 1 (WP1) is a retrospective observational study focusing on data completeness, content, and associations with social determinants of health. In contrast, Work Package 2 (WP2) adopts a retrospective cohort design under a natural experiment framework to evaluate causal relationships. This distinction will enhance the clarity of the methodological approach and ensure consistency with the study's stated objectives. Additionally, the manuscript refers to a natural experiment framework but provides limited detail on the natural variation that determines exposure (e.g., the availability or implementation of EPaCCS). | Additional text in the two work packages has been added to make clear which package is using which design.<br><br>(P11, L220)<br><br>In the first work package, a retrospective observational study, [additional new text related to another comment].<br><br>(P15, L301)<br><br>The second work package will adopt a retrospective cohort design under a natural experiment framework to evaluate the effects of EPaCCS on quality indicators of end-of-life care.<br><br>The setting and locations are demonstrated in the **Data Sources** section (P9, L204). |
| 2. Participants: While the protocol does not explicitly classify "eligibility criteria," authors define the population and data included in the analyses. The authors could strengthen the protocol by labeling these aspects as "eligibility criteria" and systematically outlining inclusion and exclusion factors in a dedicated section. Please also consider adding a subsection titled "Participants", where you can detail the eligibility criteria, the sampling method employed, and the sample size. These would enhance clarity and rigor to the protocol. | Table 2 has been amended to include the eligibility criteria, with a sentence before stating the exclusion criteria.<br><br>(P12, L249)<br><br>*Table 2* shows the mapping of the study objectives to data sources and eligibility criteria. Exclusion criteria for all data sources are participants who have chosen National Data Opt-Out and those who have withdrawn consent. |

| | |
|---|---|
| | Table 2 Mapping study objectives to data sources and eligibility to be included in analyses<br>Objectives \| Data sources and eligibility<br>Work Package 1<br><br>As stated in the manuscript (P9, L214), the sample size will be determined by the data available in the three datasets and will vary depending on the analysis. |
| 3. Analysis: Ensure that all information related to statistical analysis is relocated to the appropriate Statistical Methods subsection for better organization. To further strengthen the robustness of the analysis, it is recommended that authors include advanced methods designed to address confounding and bias, particularly in the context of natural experiments. | As our study encompasses two work packages, the manuscript does not include one Statistical Methods subsection. Rather, we decided to include an analysis subsection under the Work Package 1 section, and two analyses subsections under the Work Package 2 section outlining the ecological and individual-level analyses.<br><br>We aim to use the inverse probability of treatment weighting to address confounding and bias. This is detailed on (P16, L338). We have also included a sentence on (P16, L343) explaining how the confounders will be selected:<br><br>These confounders will be cautiously selected based on thorough discussions with our team, which includes experts in palliative care, input from the patient and public involvement group with lived experience in end-of-life care, and a comprehensive literature search. |
| **Limitations and Areas for Improvement**<br>1. Data Interoperability Challenges: • The variability in the completeness and interoperability of datasets across regions poses a risk to the comparability of results. The manuscript acknowledges this but does not adequately specify how these challenges will be mitigated during the analysis. | The sentence suggesting comparison of the results has been removed and was a legacy of an earlier protocol. The team has decided it would not be possible to compare due to the reasons the reviewer stated.<br><br>(P9, L211)<br><br>These EPaCCS are well established and contain a sufficient number of records, as illustrated in *Table 1*, to enable meaningful individual site analyses and answer our research questions.~~, as well as comparative analyses if appropriate~~. |
| 2. Regional Variability: • The differences in healthcare delivery and ACP practices across London, Leeds, and Bradford could significantly influence findings. The protocol should outline how these regional differences will be statistically accounted for to ensure valid comparisons. | This comment from the reviewer is addressed in the reply to the previous comment. |
| 3. Potential Selection Bias: • The observational nature of the study introduces inherent risks of | A sentence expanding on the selection of confounders has been added. |

| | |
|---|---|
| selection bias. Patients with EPaCCS records may systematically differ from those without in ways that influence outcomes (e.g., severity of illness, access to healthcare). While inverse probability weighting is mentioned, further details on confounder selection and balance assessment are necessary. | (P16, L343)<br><br>These confounders will be cautiously selected based on thorough discussions with our team, which includes experts in palliative care, input from the patient and public involvement group with lived experience in end-of-life care, and a comprehensive literature search.<br><br>A sentence expanding on the balance assessment and a new citation has been added.<br><br>(P16, L347)<br><br>The balance of patients' characteristics will be assessed visually and by examining the standardized mean differences.[59] |
| 4. Clarification of Sensitivity Analysis: The manuscript briefly mentions sensitivity analyses, especially regarding the COVID-19 pandemic's impact on EPaCCS documentation. More specific details on planned sensitivity analyses could clarify how the study intends to handle confounding factors. | We are considering using different approaches to identify outliers such as box plots and z-scores. Assuming imputation is considered the appropriate choice to deal with missing data (details on imputation see: P14, L266 and P17, L377) sensitivity analysis will be used to compare analysis with and without imputation.<br><br>In terms of COVID-19, we have added a sentence expanding on sensitivity analysis.<br><br>(P17, L380)<br><br>We will consider a number of approaches in terms of sensitivity analyses, which will be informed by the availability and completeness of the data. [61] Sensitivity analyses will be conducted on the cohorts identification strategy, and our choice of covariates used for estimating the propensity scores.[62] We will also consider several approaches and sensitivity analysis techniques to deal with unobserved confounding, the implementation of new policies and the effect of the COVID-19 pandemic on both the intervention and the outcomes of interest.[33 63] In terms of the COVID-19 pandemic, we will build on our previous evaluation,[51] identify the records created during the pandemic, and, if appropriate, perform a |

| | |
|---|---|
| | sensitivity analysis by potentially excluding these records to assess the impact of this period on our findings. |
| 5. Cost Analysis Details: While the study promises a cost minimization analysis, details on how costs will be attributed to specific healthcare services and modeled (e.g., inclusion of indirect costs, use of unit costs) are limited. | We aimed to clarify the nature of the cost data available within the Whole Systems Integrated Care dataset, as demonstrated in Table 1, the **'North West London dataset':**<br><br>*"Cost data is also available as patient-level costs referring to the indicative spend calculated separately for each patient for each healthcare sector. This will be used in a cost minimisation analysis of EPaCCS, and it includes primary care level, acute, community, mental health and social care costs."*<br><br>An additional sentence referring to this was added on (P15, L324) where we elaborate on the cost analysis:<br><br>The cost analysis will utilise the patient-level cost data available in WSIC.<br><br>We also illustrated there how intervention costs, formal costs, and informal care hours will be estimated. Providing further specific details at this stage is not feasible due to the limited access to the data. |
| **Suggestions for Improvement:**<br>1. Enhance Description of Data Quality Management: The protocol could benefit from a more detailed discussion on handling missing or incomplete data. The manuscript should specify the imputation methods, the thresholds for acceptable levels of missingness, and how sensitivity analyses will address potential biases introduced by missing data. | A recent study suggests that the proportion of missing data should not be used when deciding on the imputation model. Reference is: Madley-Dowd P, Hughes R, Tilling K, Heron J. The proportion of missing data should not be used to guide decisions on multiple imputation. *J Clin Epidemiol*. 2019;110:63-73. doi:10.1016/j.jclinepi.2019.02.016<br><br>Additional imputation methods details have been included. However, there is no currently agreed methodological guidance on handling these types of missingness in routine health data and we will explore different methodological approaches<br><br>(P13, L263)<br><br>Several methods will be considered that are appropriate for missing at random and not missing at random data. Though it is assumed that the missing mechanism will be missing not at random. However, no robust methods of |

| | |
|---|---|
| | imputing missingness in the independent variables with a missing not at random mechanism have been identified. |
| 2. Addressing Confounding: The protocol should provide greater detail on how confounding variables will be selected and modeled. Including a directed acyclic graph (DAG) would enhance transparency in identifying causal relationships and addressing biases. | Confounding has been addressed in reply to a previous comment. Refer to (P16, L343). In addition to this, a directed acyclic graph is planned to be published at a later stage in the research as part of the results. |
| 3. Expound on Social Determinants Analysis: The planned analysis of social determinants of health in relation to ACP and EPaCCS usage is a valuable component of the study. Expanding on the methodology for this analysis, including specific variables and adjustment methods, would improve transparency. | We have clarified which social determinants of health we are including in the analysis section for WP1.<br><br>(P13, L257)<br><br>The study will categorise the data into social determinants of health (age, gender, ethnicity, social deprivation), clinical characteristics and elements relating to ACP. The social determinants of heath have been identified from existing literature for being associated with poor health experiences and outcomes.<br><br>We have added a sentence expanding on how confounders will be selected for adjustment.<br><br>(P14, L273)<br><br>Confounders will be selected using expert knowledge, existing literature, and the completeness and availability of the data. |
| 4. Address Regional Differences: A stronger discussion of regional differences in healthcare infrastructure, population demographics, and end-of-life care preferences would enhance the study's generalizability and relevance. | Participants demographics and end-of-life care preferences (ACP) are one of the study's outputs, which will be interpreted in the discussion of the results in the context of population demographics and healthcare infrastructure. |
| 5. Sensitivity Analyses: The mention of sensitivity analyses is brief. The manuscript should elaborate on how these analyses will evaluate the robustness of findings, particularly in response to assumptions about confounders, selection bias, and the COVID-19 pandemic's impact on EPaCCS adoption. | Given the limited research in this area, we have prioritized an exploratory design that aligns more closely with our research aims.<br><br>We have expanded on our description of the selection of confounders, included in a previous comment response see: (P14, L273) and (P16, L343). |
| 6. Ethics and dissemination: While public access to datasets is reported, it is contingent on compliance with ethical and governance frameworks, ensuring secure and responsible use of sensitive health data. This aligns with best | We have added a few sentences explaining who can access the data<br><br>(P19, L440) |

| | |
|---|---|
| practices for handling healthcare data; however, greater clarity is needed regarding who will have the right to access the data or under what conditions access can be requested. | Data will not be made available to the researchers until all necessary approvals are in place. The data will only be accessible by MA, SLC, MJA, CR, PM, LS, CC, JD, and JK through Trusted Research Environments (TRE). TREs are secure research platforms that are designed to store and protect sensitive data, guaranteeing data privacy. |

2. Dr. Florbela Gonçalves's comments:

| Comments | Modifications |
|---|---|
| Thank you very much to the editors and authors for the opportunity to review this magnificent article.<br>It is very well written and innovative. I just have one question: how is data privacy guaranteed? | We would like to thank Dr. Gonçalves for their supportive feedback.<br><br>We have included a sentence explaining who will have access to the data, and how is data privacy guaranteed:<br><br>(P19, L440)<br><br>Data will not be made available to the researchers until all necessary approvals are in place. The data will only be accessible by MA, SLC, MJA, CR, PM, LS, CC, JD, and JK through Trusted Research Environments (TRE). TREs are secure research platforms that are designed to store and protect sensitive data, guaranteeing data privacy. |

## VERSION 2 - REVIEW

| | |
|---|---|
| **Reviewer** | **1** |
| **Name** | **Metallinou, Dimitra** |
| **Affiliation** | **University of West Attica, Midwifery** |
| **Date** | **04-Feb-2025** |
| **COI** | |

Dear authors,

Thank you for addressing the comments and suggestions provided in the review of your manuscript.

I appreciate the thorough revisions you have made to enhance the clarity, structure, and methodological rigor of the study protocol. It is evident that each point of feedback has

been carefully considered and appropriately incorporated into the revised version. The additional details have significantly strengthened the manuscript.

Given these revisions, I am pleased to confirm that the protocol now meets the necessary standards, and I accept the revised version for publication consideration.

I look forward to seeing the valuable contributions your study will make to palliative care research and practice.

Wishing you all the best,

Dr Metallinou Dimitra
